# Ross River Virus Immune Evasion Strategies and the Relevance to Post-viral Fatigue, and Myalgic Encephalomyelitis Onset

**DOI:** 10.3389/fmed.2021.662513

**Published:** 2021-03-24

**Authors:** Brett A. Lidbury

**Affiliations:** National Centre for Epidemiology and Population Health, Research School of Population Health, The Australian National University, Canberra, ACT, Australia

**Keywords:** myalgic encephalomyelitis, fatigue, virus, inflammation, immunity, cytokines, mitochondria

## Abstract

Ross River virus (RRV) is an endemic Australian arbovirus, and member of the Alphavirus family that also includes Chikungunya virus (CHIK). RRV is responsible for the highest prevalence of human disease cases associated with mosquito-borne transmission in Australia, and has long been a leading suspect in cases of post-viral fatigue syndromes, with extrapolation of this link to Myalgic Encephalomyelitis (ME). Research into RRV pathogenesis has revealed a number of immune evasion strategies, impressive for a virus with a genome size of 12 kb (plus strand RNA), which resonate with insights into viral pathogenesis broadly. Drawing from observations on RRV immune evasion, mechanisms of relevance to long term idiopathic fatigue are featured as a perspective on infection and eventual ME symptoms, which include considerations of; (1) selective pro-inflammatory gene suppression post antibody-dependent enhancement (ADE) of RRV infection, (2) Evidence from other virus families of immune disruption and evasion post-ADE, and (3) how virally-driven immune evasion may impact on mitochondrial function via target of rapamycin (TOR) complexes. In light of these RRV measures to counter the host immune - inflammatory responses, links to recent discoveries explaining cellular, immune and metabolomic markers of ME will be explored and discussed, with the implications for long-COVID post SARS-CoV-2 also considered. Compelling issues on the connections between virally-induced alterations in cytokine expression, for example, will be of particular interest in light of energy pathways, and how these perturbations manifest clinically.

## Introduction

The history of Myalgic Encephalomyelitis (ME) features outbreaks as defining events, suggesting an infectious etiology behind the cluster of symptoms experienced by affected individuals (1, 2). Outbreak examples include Akureyri disease (Iceland - 1940s), Royal-Free Hospital London (UK - 1950s), “Tapanui Flu” (New Zealand - 1980s), and Lake Tahoe (USA - 1980s), with the description of “flu-like” symptoms common among patients (3, 4). While respiratory viruses were obvious candidates given this history, other virus families have been linked to ME (5), as well as the intra-cellular bacterial agent of Q-Fever, *Coxiella burnetti* (6, 7).

Viruses rely upon the host-cell machinery to replicate, and knowledge of these processes for diverse virus families is well-established (8). Appreciation of the manipulation of host-immunity by viruses has emerged during the previous three decades (9, 10), with early insights gained from large genome DNA viruses, for example poxviruses and herpesviruses, which often involved cytokine ligand or receptor mimicry to evade host-immune responses (11–15). RNA viruses also have evolved ingenious strategies for immune evasion (16, 17), with examples including members of the flavivirus and coronavirus families (18–21) that are responsible for disease on a global scale. From an evolutionary perspective, the author has previously theorized that RNA viruses rely upon “erroneous replication” strategies to avoid destruction by the host, but at the potential cost to the virus of killing the host and impeding the propagation of viral progeny (16).

The perspective presented here centers on the endemic Australian virus, Ross River (RRV), a positive-strand RNA (12 kb) Old World Alphavirus. RRV is transmitted by mosquitos and responsible for Ross River virus disease (RRVD), which is defined by lethargy, myalgia, rash and polyarthritis, as well as post-viral syndrome (22, 23). RRV is suspected of being a microbial precursor to Australian ME cases, along with Epstein-Barr Virus (EBV) and *C. burnetti* (7).

How infection leads to ME is the subject of the perspective presented herein, with RRV as the primary ME-linked virus example. Of particular interest are the observations on the dysregulation of cytokine expression in macrophages post RRV-ADE (antibody-dependent enhancement) infection, and thus inflammatory responses by the host, as well as the impact on mitochondrial function. A perspective on how these interacting features result in the ultimate clinical manifestations of long-term fatigue, post-exertional malaise and other symptom clusters (2) is presented herein.

Consideration of these questions has contemporary urgency due to the emergence of “long-COVID,” which for some patients resembles ME once recovered from the acute SARS-CoV-2 infection (24).

### Perspective Context

The concepts investigated to formulate the perspective presented are of particular contemporary importance, since at the time of writing, the world is confronting the SARS-CoV-2 (COVID-19) pandemic that has raised issues related to:

- Vaccine safety in the context of antibody-dependent enhancement (ADE) of virus infection, and
- The emergence of cases of “long-(haul)-COVID,” which in some present symptoms identical to ME.

Vaccines are not the primary focus here, but understanding ADE as an avenue of immune evasion, and thereafter manipulation of host immune - inflammatory responses by the virus, raise pertinent questions linked to the eventual development of idiopathic fatigue in some individuals post-acute virus infection. The concept of “cytokine storm” is well-recognized for COVID-19 and other diseases, but in other cases impacts of immune manipulation are maybe subtler? And what do these events mean for the regulation mitochondrial function and energy production if the virus is assisted by ADE?

### Antibody-Dependent Enhancement

Hawkes first reported ADE of virus infection in 1964, observed for members of the *Togavirus* Family as classified at the time (25). The studies focussed on Class A and B Togaviruses, with Getah (Togavirus - *Alphaviridae*) Murray Valley Encephalitis, West Nile and “Japanese Encephalitis viruses” (Flaviruses - *Flaviridae*) displaying up to 12-fold growth enhancement in chick embryo fibroblast (CEF) cultures, and on chorioallantoic membranes, with the effect only seen with antibodies raised in “domestic fowls,” not from other species. Further investigations revealed that the enhancing properties were specific to the IgG fraction of the anti-serum that enhanced virus growth (26).

Presciently, once antibody was identified as the enhancing factor, the authors stated; *Another possibility that should be considered is that the enhancing antibody is taken into the cell along with the virus and influences subsequent intracellular events. These problems can only be answered by further studies of the interactions between complexes of virus and antibody and susceptible cells* (26).

Subsequent studies conducted over the 1970s−80s established *in vitro* ADE for the global pathogen Dengue virus (DEN), as well as identified the role for Fc-Receptor (Fc-R) engagement in ADE via studies with other flaviviruses (27–29). By the 1990s, ADE was recognized as a factor in severe DEN disease (Haemorrhagic Fever, Shock Syndrome), on subsequent infection with a DEN serotype different to the original case (30), which has also frustrated attempts to develop a DEN vaccine (31). Many virus families have been observed as displaying enhanced *in vitro* growth post-infection due to ADE, but the impact *in vivo* and on disease manifestation are not currently well-understood (32–34).

While ADE was observed for the close RRV relative, Getah virus, during Hawkes's original ADE observations in 1964, RRV-ADE was not reported until the 1990s (35). As found for earlier examples, RRV-ADE was demonstrated *in vitro* for monocytes and macrophages, including the macrophage cell line RAW 264.7, which was later central to the elucidation of molecular mechanisms post-ADE entry.

### The Disruption of Macrophage Pro-inflammatory Responses as an RRV-ADE Mechanism

While enhanced virus uptake helped explain ADE mechanism (28), the “… *subsequent intracellular events* …” suggested by Hawkes and Lafferty were eventually revealed by *in vitro* models of RRV infection using RAW 264.7 macrophages. The important intracellular events were linked to the regulation of pro- and anti-inflammatory cytokines, at the level of the transcriptional machinery. While the impact on early virus growth is clear, it must be assumed that the dysregulation of cytokines during an innate response also has implications for longer term immunity, and perhaps the vigor of subsequent inflammation that follows infection.

The RRV *in vitro* models showed a clear disruption of expression for TNF, NOS2, IFN-β, IP-10 that involved the temporary post-ADE ablation of STAT-1 (ISGF3 and AAF), IRF-1 and NK-κB transcriptional complexes, which occurred at the same time as enhanced RRV growth. As well as the downregulation of proinflammatory - antiviral gene expression, IL-10 expression was significantly increased, as was the transcription factor Sp1 (36, 37). Similar patterns were also detected in a flavivirus (DEN) (38–42) and an arterivirus (PRRSV) (43–46). Post-ADE IL-12 and IFN-γ suppression was also observed for DEN, as well as concomitant impacts on the associated transcription factors (e.g., STAT, IRF), while IL-10 expression was similarly unaffected or increased (Table 1).

**Table 1:** Examples of ADE-mediated virus infections and the impact on subsequent cellular pathways and cytokine expression.

**Virus family (*Genus*)**	**Examples (*Disease*)**	**Cell examples - intracellular events post-ADE: cytokines impacted**	**References**
Togaviridae (*Alphaviridae*)	Ross River (RRV) - (*Lethargy, Myalgia, Polyarthritis*)	Monocyte, Macrophage (RAW 264) - Post ADE suppression of TNF, NOS2, IFN-β, IP-10 expression via IRF-1, NF-κB, STAT-1 complex (ISGF3, AAF) ablation; Increased IL-10 expression (mRNA, Protein); Sp1 elevated	(37) (36)
Flaviviridae (*Flavivirus*)	Dengue - (*Fever, Shock, Myalgia, Hemorrhage*)	Monocyte, Macrophage - Post ADE suppression of IL-12, IFN-γ TNF; Increased IL-6, IL-10 (0-5 days p.i) with pSTAT-1, IRF-1 impacted; Increased IL-10 expression SOCS3, Syk-regulated; Early NOS2 via RLR-MAVS (without IFN); Autophagy role (ATG5) in IFN restriction; Early Syk - ERK1/2 IL-1β stimulation independent of DEN replication. Mast cell/Basophil - Post-ADE (72 hrs) significant increases for IL-1β, IL-6, not GM-CSF	(47) (40) (39) (42) (41) (38) (48)
Arteriviridae *(Arterivirus)*	^*^ Porcine Reproductive & Respiratory Syndrome Virus (PRRSV) - (*Abortion, Respiratory disease in pigs*)	Alveolar macrophage - ADE-mediating viral epitopes mapped to N and GP5 proteins; ADE via FcγRI, FcγRIIb, FcγRIII; IFN-α, TNF-α expression decreased; IL-10 increased (mRNA, protein); IRF-1 IRF-3, NF-κB disrupted	(43) (45) (46) (44)
*Filoviridae* (Ebolavirus)	Ebola (*Hemorrhage, Shock*)	Granulocyte blasts (K562 cells) FcγRII, C1q-mediated ADE (via CR); FcγRIIa signaling via Src family protein tyrosine kinases (PTKs); Endosome uptake (phago-pinocytosis), Src phosphorylation	(49) (50) (51)
*Coronaviridae* (Beta-coronavirus)	SARS-CoV (1, 2) (Acute respiratory disease, Post-acute long-term fatigue)	Monocyte/Macrophage (THP-1; CD68^±^/CD14^±^ PBMC) - FcγRII required (intracellular domain); ADE infection achieved, but abortive in the longer term; IFN-α/β, MCP-1, IP-10, TNF, MIP-1 mRNA expression not altered post-ADE (1–72 h p.i.)	(52) (53) (54)

*Virus details - NCBI Taxonomy Browser (www.ncbi.nlm.nih.gov/Taxonomy/Browser/wwwtax.cgi)*.

* *Recently suggested nomenclature - Beta-arterivirus suid 1*.

*For the history, and scientific reviews of ADE across virus families, see Taylor et al. (32), Tirado and Yoon (33), and Porterfield (34)*.

Knowledge of intracellular events post-ADE has been assisted through understanding the biology of Fc-gamma-Receptors (FcγR), including the identity of FcγR classes, the affinity of IgG (or complex) receptor binding and linked intracellular activating (e.g., FcγRIIa ➢ ITAM) or inhibitory (FcγRIIb ➢ ITIM) pathways (55). For the inhibitory action of FcγRIIb, phosphatases are recruited to the ITIM domain post receptor cross-linking (SRC family kinases), and ultimately leads from PIP_3_ to PIP_2_ conversion via hydrolysis. PIP_3_ is a cell surface receptor linked second messenger synthesized from PI3K isoforms, involved in a range of cellular functions post ligand engagement. Of potential relevance to the role of ADE-mediated suppression of inflammatory pathways after FcγRIIb interaction, mouse models of disease have demonstrated that the inhibition of PI3Ks diminishes the severity of inflammation (56).

DEN has provided a strong focus into the intracellular consequences post-ADE infection, describing other intracellular mechanisms beyond those originally identified by RRV. These extend to type I IFN restriction via autophagy, SOCS3 and *Syk*-regulated pathways (Table 1). DEN-ADE and cytokine expression changes have been also reported for mast cells (48). Ebola virus ADE showed interesting FcγRIIa signaling pathways, without details on cytokine expression (49–51), although Ebola has been shown to alter cytokine expression without ADE via secreted viral glycoprotein (57).

SARS-CoV is an ADE virus, but with a difference (Table 1). While ADE for FcR bearing cells was observed, longer term infection was abortive, and the post-ADE mRNA expression profiles for IFN-α/β, MCP-1, IP-10, TNF, MIP-1 were not altered (52–54). Whether ADE is a factor in SARS-CoV pathology, or poses a threat post-vaccination, are currently being debated and assessed (58, 59). However, there is broad consensus that “cytokine storm,” which can be understood as a gross dysregulation of appropriate cytokine responses leading to hyperinflammation, is a factor in disease (e.g., acute respiratory distress symptom). The link of SARS-CoV to ME is the recognition of “long-COVID,” which shares symptoms such as long-term unexplained fatigue, “brain fog,” pain and so on (24, 60), and as such provides a connection between an acute virus infection and long-term sequelae, as has been suspected in ME for decades. Definitive cytokine profiles for long-COVID have not been determined as yet, and drawing from the ME experience of establishing cytokine profiles for long term illness is not helpful due to the lack of consistency and validation by larger studies, although TGF-β has attracted some interest (61, 62).

### Linking Inflammation, Cytokine Expression, and Mitochondrial Function

The core ME symptoms of long-term fatigue and post-exertional malaise (PEM) logically point to bioenergetic pathways, metabolomics and mitochondrial function, which have attracted biomedical research attention over the previous 10–15 years (63–66). Very recently, studies by Missailidis et al. (67, 68) have investigated mitochondrial function in immortalized *ex vivo* lymphoblasts collected from ME patients. Among a number of observations, Complex V rate of ATP synthesis was significantly reduced compared to healthy control lymphoblasts, with a statistical difference also found for lymphocyte death rate, and with chronic TORC I (TOR-Complex I) hyperactivation in ME lymphoblasts observed (suggesting compensatory activity via the upregulation of proteins required for oxidative phosphorylation and general mitochondrial function). TORC signaling is critical for stress sensing, cell growth and energetics, hence important to *homeostasis* and life span in general, with implications for disease if altered (69, 70).

Infection and the virally-induced disruption of cytokine signaling impacts homeostasis, and evidence exists to support mTOR - STAT signaling interactions in the context of immunity, including IL-10 expression (71). Therefore, chronically upregulated TORC signaling, as recently observed (67), may be linked to upsets in STAT, or vice versa. Inflammation impacts TOR function, and of relevance to the disruption to cytokine expression in macrophages post RRV-ADE, mTORC2 regulation is necessary for IFN-stimulated genes (ISGs) (37, 71).

The disturbance of homeostasis is an obvious result of infection, with the advent in some patients of severe illness due to cytokine storm, which represents a serious disequilibrium outcome due to the compromise of normal inflammation regulation processes. In discussing pathology, it is often forgotten that immune responses are also required for cellular and tissue repair, which involve the pleiotropic nature of some cytokines that are normally associated with inflammation, like TNF (72, 73), in addition to other cytokine families with primary roles in healing, for example, the transforming growth factor (TGF) family. In fact, a member of the TGF super family, Activin B, has been identified in serum from ME patients as significantly different when compared to healthy volunteers (74). Activin proteins have many physiological roles, including repair, pro-, and anti-inflammatory functions (75).

In answering questions on why some individuals develop severe symptoms post-infection, and then in a proportion of cases intractable ME or long-COVID, while others display no short or long terms health impacts, surely is connected to individual differences in the regulation of the interactions discussed above. And within this milieu, the TOR family of proteins sit at the interface between the regulation of inflammation - immunity post-infection, and energy regulation both at the mitochondrial level, and associated pathways required for carbohydrate, lipid and amino acid catabolism.

## Discussion

While ADE is not the primary focus here, past investigations of ADE mechanism have identified a range of cellular pathways manipulated by viruses that may alter future cellular function, potentially leading to a long-term disturbance of homeostasis, which can lead to chronic alterations in mitochondrial function and energy regulation, ultimately manifesting as multi-system disease. By linking patterns of post-infection cytokine dysregulation with observations on chronically increased TOR protein activity in cells from ME patients, the interface of TOR and inflammatory pathways is recommended as a topic for deeper investigation. Delving into these cellular processes will contribute insights into the mystery of why a virus infection can lead to a chronic health condition like ME in some individuals (estimated to be 11% in Australia) (7). The emergence of long-COVID brings a new urgency to these questions.

Of course, viruses do not need ADE entry to manipulate host immune-inflammatory responses to infection [(5) - includes explanations of virus-associated disruption of mitochondrial function, impact on immune cells, and discussion of infection and ME pathogenesis], but the impact associated with the expansion of cellular range to Fc-Receptor (FcR) or Complement-Receptor (CR) bearing cells, not normally permissive to infection, requires consideration. The strong disruption, and at times ablation of antiviral and inflammatory pathways, must have downstream impacts on later innate immune functions and the formation of adaptive immunity, particularly with the higher viral load allowing more FcR and CR cells to become infected, and their functions similarly impacted.

## Conclusions

The history of ME features regular “outbreaks,” which have been associated with virus infections. At the time of writing, the COVID (SARS-CoV-2) pandemic has revealed a sub-population of recovered patients who have developed long-term symptoms that resemble classic ME. Therefore, a perspective is presented herein that aims to link the viral manipulation of host antiviral and inflammatory-immune responses to mitochondrial function, with TOR proteins as the critical interface between deranged cytokine expression and energy regulation. Established for many virus families (Table 1), ADE post-infection is the particular perspective focus. ADE currently has renewed interest in relation to potential COVID vaccine safety, but in a more general context also raises questions on ME pathogenesis due to the dramatic consequences for immediate antiviral defenses, later innate immune responses, and thereafter guidance from ADE-impacted cells (e.g., antigen-presenting cells) for the formation of an appropriate adaptive immune response to support long term homeostasis.

The unraveling of the interactions between the viral manipulation of cells, bioenergetics and mitochondrial function will reveal the differences, at a cellular level, to explain why some individuals go on to develop chronic long-term health challenges like ME or long-COVID, while others do not.

## Author Contributions

The conception, research, analyses, and writing of this manuscript were all conducted by BAL.

## Conflict of Interest

The author declares that the research was conducted in the absence of any commercial or financial relationships that could be construed as a potential conflict of interest.

## Abbreviations

AAF: IFN-α-activated factor
ADE: Antibody-Dependent Enhancement
ATG5: Autophagy-related 5
COVID: Coronavirus Disease
CR: Complement Receptor
DEN: Dengue virus
EBV: Epstein-Barr virus
FcR: Fc Receptor
GM-CSF: Granulocyte-macrophage colony-stimulating factor
IFN: interferon
IL: Interleukin
IP-10: IFN-inducible protein 10
IRF: IFN-regulatory factor
ISGF3: IFN-stimulated gene factor 3
MIP-1: macrophage inflammatory protein-1
MCP-1: Monocyte chemoattractant protein-1
ME: Myalgic Encephalomyelitis
NF-κB: nuclear factor kappa B
NOS2: nitric-oxide synthase 2- inducible NOS
p.i.: post-infection
PIP_2_: Phosphatidylinositol 4,5-biphosphate
PIP_3_: Phosphatidylinositol 3,4,5-triphosphate
PRRSV: Porcine Reproductive and Respiratory Syndrome Virus
PTKs: protein tyrosine kinases
RIG-1: Retinoic acid Inducible Gene-I
RLRs: RIG-I-Like Receptors
RLR-MAVS: RNA-triggered RLR-mitochondrial antiviral signaling protein
RRV: Ross River virus
SARS-CoV: Severe Acute Respiratory Syndrome-Coronavirus
SOCS3: Suppressor of Cytokine Signaling 3
Sp1: Specificity protein 1
STAT: Signal Transducer and Activator of Transcription
Syk: Spleen Tyrosine Kinase
TOR: Target of Rapamycin
mTOR: mechanistic TOR
TORC: TOR Complex
TNF: Tumor Necrosis Factor
TGF: transforming growth factor.
